# Disease Prediction Using Machine Learning on Smartphone-Based Eye, Skin, and Voice Data: Scoping Review

**DOI:** 10.2196/59094

**Published:** 2025-03-25

**Authors:** Research Dawadi, Mai Inoue, Jie Ting Tay, Agustin Martin-Morales, Thien Vu, Michihiro Araki

**Affiliations:** 1 Artificial Intelligence Center for Health and Biomedical Research National Institutes of Biomedical Innovation, Health and Nutrition Osaka Japan; 2 National Cerebral and Cardiovascular Center Osaka Japan; 3 Faculty of Medicine Graduate School of Medicine Kyoto University Kyoto Japan; 4 Graduate School of Science, Technology and Innovation Kobe University Kobe Japan

**Keywords:** literature review, machine learning, smartphone, health diagnosis

## Abstract

**Background:**

The application of machine learning methods to data generated by ubiquitous devices like smartphones presents an opportunity to enhance the quality of health care and diagnostics. Smartphones are ideal for gathering data easily, providing quick feedback on diagnoses, and proposing interventions for health improvement.

**Objective:**

We reviewed the existing literature to gather studies that have used machine learning models with smartphone-derived data for the prediction and diagnosis of health anomalies. We divided the studies into those that used machine learning models by conducting experiments to retrieve data and predict diseases, and those that used machine learning models on publicly available databases. The details of databases, experiments, and machine learning models are intended to help researchers working in the fields of machine learning and artificial intelligence in the health care domain. Researchers can use the information to design their experiments or determine the databases they could analyze.

**Methods:**

A comprehensive search of the PubMed and IEEE Xplore databases was conducted, and an in-house keyword screening method was used to filter the articles based on the content of their titles and abstracts. Subsequently, studies related to the 3 areas of voice, skin, and eye were selected and analyzed based on how data for machine learning models were extracted (ie, the use of publicly available databases or through experiments). The machine learning methods used in each study were also noted.

**Results:**

A total of 49 studies were identified as being relevant to the topic of interest, and among these studies, there were 31 different databases and 24 different machine learning methods.

**Conclusions:**

The results provide a better understanding of how smartphone data are collected for predicting different diseases and what kinds of machine learning methods are used on these data. Similarly, publicly available databases having smartphone-based data that can be used for the diagnosis of various diseases have been presented. Our screening method could be used or improved in future studies, and our findings could be used as a reference to conduct similar studies, experiments, or statistical analyses.

## Introduction

The use of machine learning for medical diagnosis is steadily growing. This can be attributed primarily to the availability of numerous health data as well as improvements in the classification and recognition systems used in disease diagnosis. The health care industry produces an abundance of health-related data [1], which can be used to create machine learning models. These models can be used for diagnosing and predicting a variety of diseases, including breast cancer, heart diseases, and diabetes [2,3]. The prediction of these diseases is dependent on many factors according to the focus on different features (biomarkers) [4]. The application of machine learning methods helps classify and diagnose diseases in an easier way [1], and these diagnoses can help medical experts in the early detection of fatal diseases and therefore increase the quality of health care and the survival rate of patients significantly [1,2,4,5].

Machine learning methods and their applications are not limited to particular types of data and thus have been used in a variety of areas, such as detecting spontaneous abortion [6], identifying complex patterns in brain data [7], and improving diagnostic accuracy and identifying faults in axial pumps [8]. To diagnose and predict different diseases, machine learning methods have also been applied to data obtained from experiments by using publicly available datasets, such as the UCI machine learning library [2], National Health and Nutrition Examination Survey (NHANES) [3,6], traumatic brain injury (TBI) [4], and SUITA datasets [9]. Similarly, numerous smartphone-based health care apps have been developed to help both health care officials and the general population with regard to their health-related concerns. The apps developed can be broadly divided into 3 specific user groups: health care professionals, medical/nursing students, and patients [10]. The purpose of such apps covers a wide range of areas, such as disease diagnosis, drug reference, medical education, clinical communication, and fall detection. However, all iOS- or Android-based apps developed for health care purposes have not been discussed in the literature [10].

A literature review is a systematic way of collecting studies relevant to a research topic, assessing the methodologies and results of the studies, and making recommendations for improvements if necessary [11]. In the health care domain, the implementation of literature reviews has been considered important for conducting further research and developing guidelines for clinical practice [12]. Literature studies, such as umbrella reviews, have been conducted to study the management of the information of patients, such as those with cancer, and how their records are handled [13]. Similarly, literature-based studies have investigated the evidence of leadership in nursing [14]. Uddin et al [15] found a total of 48 literature studies that dealt with disease prediction using various supervised machine learning algorithms and attributed the rise in the use of machine learning for health prediction to the wide adoption of computer-based technologies in the health sector and to the availability of large health-related databases. 

The ubiquity of smartphones makes them a convenient tool to gather various health-related data, particularly as smartphones are equipped with various sensors that are able to track and gather different health-related information [16]. However, there is a lack of research on studies involving the adoption of smartphones for disease prediction using machine learning methods and identifying the types of experiments conducted, databases utilized, and machine learning methods used. With that in mind, in this paper, we aim to conduct a scoping review by assessing research papers from repositories, such as PubMed and IEEE Xplore, which have used machine learning methods with smartphone-derived data to predict diseases related to the eyes, skin, and voice, and from databases available for public use. We aim to answer the following important research questions:

1. What are the databases available for eye-, skin-, and voice-related diseases?
2. What are the machine learning models used in such studies?
3. How the data are collected using smartphones?

The rest of the paper is organized as follows: we explain how we gathered, screened, and analyzed the literature in the methods section; present the results of our study in the results section; and finally discuss the results and clarify how the results correspond to our research questions in the discussion section.

## Methods

### Overview

We describe in detail the procedures undertaken for conducting the scoping review, with inspiration taken from the guidelines provided by Mak and Thomas [17]. After deciding on the topic of research, we identified the steps to be taken for the literature review as follows:

1. Search criteria
2. Literature assembly
3. Study selection
4. Research questions
5. Inclusion and exclusion criteria
6. Full-text paper assessment

### Search Criteria

Numerous studies have conducted literature reviews to assess the use of machine learning for disease prediction. We formulated the following search string using a combination of different words related to topics, such as smartphone, smartwatch, machine learning, health, and medicine, to search different electronic databases: ((ML OR machine learning) AND (health* OR medic* OR disease) AND (smartphone OR smart phone OR smartwatch OR smart watch OR smart devices)).

Before finalizing the search string, we experimented with many combinations, including different variations of specific keywords and symbols, such as “*,” to cover a wider area and maximize the results. 

### Literature Assembly

We applied the search string to different databases and narrowed the databases to PubMed [18] and IEEE Xplore [19]. The search results from other databases produced a very high number of results that included unnecessary papers from disciplines unrelated to our topic of interest. When we applied the same search string, ACM Digital Library had about 24,000 results, ProQuest had about 125,000 results, and Google Scholar had more than 1 million results. Furthermore, in Science Direct, our search string did not produce any results owing to the use of Boolean connectors. We concluded that the search of PubMed and IEEE Xplore was enough to obtain papers related to research in technology, engineering, and biomedical sciences.

The results from each of the databases were then exported to an external file. To convert the results from the 2 databases into a single file, including the titles and abstracts, we used Mendeley [20] and Zotero [21]. The file, which contained a total of 2390 papers, was then screened in the Jupyter Notebook environment using Python (version 3.7.17) [22].

### Study Selection

For refining the collected papers, we used a title screening method [23] to filter out papers that might not be of direct relation to our research topic. We created a list of keywords that match the research topic, screened the titles of all papers, and filtered out all papers that did not contain any of the following keywords: machine, artificial, smartphone, disease, mobile, health, healthcare, wearable, model, features, and training.

The identified papers at this point covered a wide variety of diseases and health areas. Using the keyword identification method, we tried to find the distribution of different diseases in the papers based on the 5 senses [24]. We first determined the frequency of keywords related to the 5 senses in the titles of the collected papers by using the following keywords: eye, eyesight, vision, audio, voice, vocal, nasal, nose, hearing, ear, touch, feel, face, skin and dermatology.

The result for the frequency of the keywords in the titles can be seen in Table 1. We then merged the keywords with their respective senses and assembled the papers into the following 6 categories: ear, eye, nose, touch, skin, and audio. We determined the total distribution of the papers, as shown in Table 2. We replicated the procedure to determine the frequency of health categories (Table 3) and their distribution (Table 4) in the abstracts of the collected papers.

**Table 1:** Health categories in titles.

Health care area	Number of matches
Eye	12
Eyesight	0
Vision	13
Audio	15
Voice	16
Vocal	5
Nasal	0
Nose	2
Hearing	5
Ear	2
Touch	6
Feel	0
Face	13
Skin	19
Dermatology	2

**Table 2:** Distribution of health categories in titles.

Category	Distribution, %
Voice	32.7
Nose	1.8
Ear	6.4
Eye	22.7
Touch	5.5
Skin	30.9

**Table 3:** Health categories in abstracts.

Health care area	Number of matches
Eye	108
Eyesight	1
Vision	142
Audio	106
Voice	141
Vocal	24
Nasal	4
Nose	18
Hearing	28
Ear	30
Touch	32
Feel	5
Face	130
Skin	162
Dermatology	20

**Table 4:** Distribution of health categories in abstracts.

Category	Distribution, %
Voice	28.5
Nose	2.3
Ear	6.1
Eye	26.4
Touch	3.9
Skin	32.8

### Research Questions

Based on the results from Tables 1-4, we identified the following 3 categories with the highest distribution of papers: eye, skin, and voice, and formulated the following research questions:

1. What are the databases available for eye, skin, and voice analysis?
2. What are the machine learning models used for eye, skin, and voice analysis?
3. How are the data collected from smartphones?

The keyword screening method [23] was applied to the titles of 2390 papers, which resulted in the successful screening of 2352 papers. In the next step, we screened the abstracts of the papers to distinguish papers related to each of the 3 topics (eye, skin, and voice) by using relevant keywords.

### Inclusion and Exclusion Criteria

The primary inclusion criterion was that the study should perform an experiment or use a database involving data obtained by using smartphones. Some studies conduct experiments themselves to gather data from participants, while others use publicly available datasets. We divided the studies based on this distinction (experiments and databases). This information can help researchers determine if they want to conduct similar experiments or simply use publicly available databases.

Since the search terms specified the use of both smartphones and machine learning methods, it reduced the probability of obtaining literature results related to topics other than disease prediction among humans. The other criteria for the articles were that they should be in the English language and should be available for full-text viewing. Studies that involved data collection with external devices other than smartphones and those that used only smartwatches and not smartphones were excluded. Furthermore, studies that were literature reviews were not included in the final analysis.

### Full-Text Assessment

The inclusion and exclusion criteria were applied to 217 papers available after title and abstract screening. After assessment of these papers, there were 8, 14, and 38 studies related to the skin, eyes, and voice, respectively. We performed full-text analysis of these papers to extract the desired information.

## Results

### Overview

**Figure 1:**
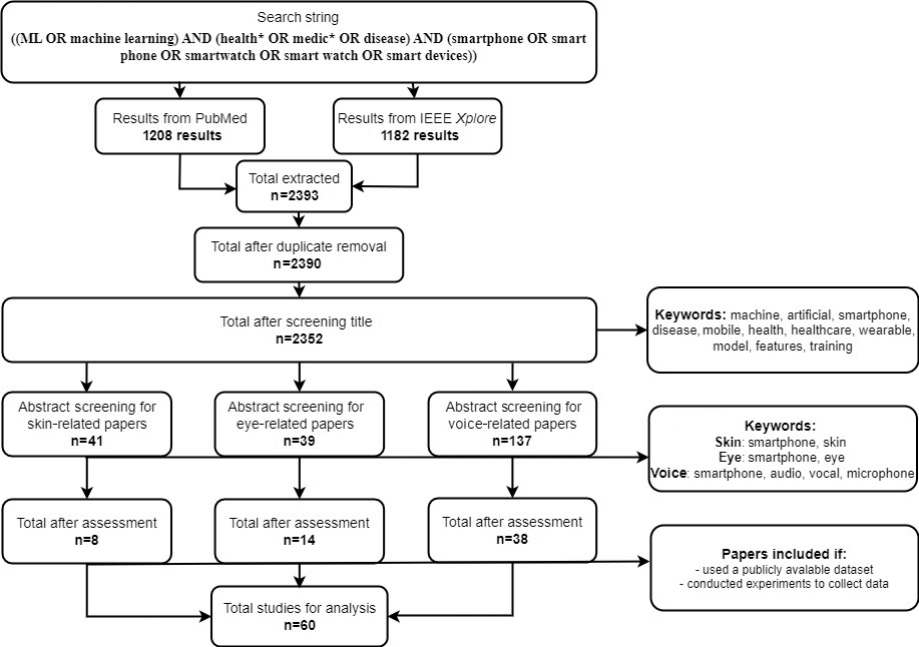
Flow diagram for identifying relevant literature.

We explain the analysis of papers that were extracted and report about the databases used, experiments conducted, and machine learning methods used. The steps and results of our review process can be seen in Figure 1.

### Research on Voice

Owing to the recent global pandemic, research on the analysis of speech for either cough or COVID-19 has grown [25]. Apart from that, the analysis of audio has a wide range of applications from the prediction of emotional stress [26] and the detection of diseases, such as Parkinson disease [27], to the detection of tourist emotions for spot recommendation [28].

#### Studies Conducted Using Databases

A cough-based COVID-19 detection model was created using more than 25,000 cough recordings from the CoughVid dataset [25]. The dataset was created through recordings via a web interface that could be accessed by a personal computer or a smartphone, and the prediction was made using a stack ensemble classifier consisting of machine learning methods, such as decision tree (DT), random forest (RF), k-nearest neighbor (KNN), and extreme gradient boosting (XGBoost).

Since datasets containing the voices of COVID-19–affected patients were not in abundance, datasets with recordings of cough sounds along with sneezing, speech, and nonvocal audio were used to pretrain the classifier [29]. Brooklyn and Wallacedene datasets used for the training were created using an external microphone, while datasets, such as TASK, were created using an external microphone along with a smartphone. It is very likely that smartphones were used to create datasets, such as the Google audio dataset and Freesound, which consist of audio from more than 1.8 million YouTube videos. Similarly, the Librispeech dataset consists of audio from 56 speakers who may or may not have used smartphones. For the classification and testing of the model, 3 datasets, namely Coswara, ComparE, and Sarcos, were used by applying machine learning methods, such as convolutional neural network (CNN), long short-term memory (LSTM), and RestNet50. All 3 datasets were created with the recordings of the cough of participants. ComparE and Coswara consist of additional speech sounds, with the Coswara dataset also including breathing sounds. The data acquisition method was web-based, and thus, smartphones could have been used for recording such audio data.

The Coswara dataset, with recordings of over 1600 participants, was created by collecting breathing, coughing, and voice sounds, using the microphones of smartphones via an interactive website application. With a combination of hand-crafted features and deep-activated features learned through model training, a deep learning framework was proposed and studied by using the recordings of 240 participants from the Coswara dataset (120 participants were identified as positive for COVID-19) [30].

The dataset created in the mPower study [31], which was conducted for the detection of Parkinson disease using audio data, has been used in various other studies [27,28]. The dataset was divided into training and test sets, and 2 classifiers, namely support vector machine (SVM) and RF, were applied to compare 6 cross-validation techniques [32]. Similarly, a desktop application, PD Predict, that records audio and makes predictions was created using the mPower dataset [33]. Two machine learning classifiers were used: gradient boosting classifier (GBC) pipeline with Lasso (gbcpl) and GBC pipeline with ElasticNet (gbcpen).

Moreover, using a dataset containing 18,210 recordings from the mPower study [31], a Parkinson disease prediction model was created through 4 classifiers: SVM, KNN, RF, and XGBoost [32]. Another Parkinson disease prediction model was created with 2 databases: PC-GITA and Vishwanathan [34] by using SVM. For creating the PC-GITA dataset, a smartphone was used to record 100 Columbian-Spanish speakers, among whom 50% had Parkinson disease. Similarly, 46 participants, among whom 24 were diagnosed with Parkinson disease, were used to create the Vishwanathan dataset, which consists of recordings of utterances of the alphabets “a,” “u,” and “m.”

Five machine learning models, namely logistic regression (LR), RF, XGBoost, CatBoost, and Multilayer, were used to predict the emotional state of participants [35]. The dataset Extrasensory was used for training the model. The dataset was created using data from smartphones and smartwatches. Contextual data, such as location, phone state, accelerometer data, and light and temperature data, and emotional state information (disclosure of emotion at different intervals using a smartphone app) were collected.

#### Studies Conducted Through Experiments

A total of 1513 subjects above 50 years of age, including healthy subjects and subjects who were diagnosed with Parkinson disease, used a smartphone app to complete daily surveys and 4 activities intended to test the presence or effect of Parkinson disease [31]. The activities included tapping (tap 2 buttons alternatively), walking (walk in a straight line for 20 steps and back in the same route), voice (10-second utterance of the “aaah” sound), and memory (recall the order of illumination of flowers shown in the app). The data related to the accelerometer, gyroscope, touchscreen, and microphone were then collected to test the results of these activities. LR, RF, deep neural network (DNN), and CNN were used separately and as multi-layer classifiers for model creation and verification.

An Android-based smartphone app was developed to record 5 activities (voice, finger tapping, gait, balance, and reaction time) in 129 participants, including subjects who were healthy and those who were diagnosed with Parkinson disease, in order to study the effects of the disease [26]. Disease severity score learning (DSSL), a rank-based machine learning algorithm scaled from 0 to 100 (higher numbers reflect increasing severity of the disease), was used to show the results. In another study, 2 vocal tasks of patients diagnosed with Parkinson disease were recorded in a soundproof booth: one in which the participants spoke the vowel “a” for 5 seconds, and another in which the participants spoke a sentence in their native Lithuanian language [36]. The recordings were conducted using both an external microphone and a smartphone, and the model was created using RF.

In another study, 237 participants diagnosed with Parkinson disease performed 7 smartphone-based tests, such as pronouncing “aaah” on the smartphone for as long as possible, pressing a button on the screen if it appears, pressing 2 alternate buttons on the screen, and holding the phone with their hand at rest or outstretched. Their balance and gait were also analyzed from the position of the smartphone [37]. The data obtained from the smartphone were used to train the machine learning algorithm using RF. The dataset was divided into training and test sets, and the prediction accuracy was tested using 10-fold cross-validation and leave-one-out cross-validation.

In addition to voice, facial features can be used for the detection of Parkinson disease [38]. Using both facial and audio data from 371 participants, among whom 186 were diagnosed with Parkinson disease, it was observed that early-stage detection of Parkinson disease is possible by combining both data. Participants were asked to read an article containing 500 words, and an iPhone was used to record both audio and video. DT, KNN, SVM, LR, naive Bayes, RF, LR, gradient boosting (GBoost), adaptive boosting (AdaBoost), and light gradient boosting (LGBoost) were compared to assess their performance in terms of accuracy, precision, recall, *F*_1_-score, and area under the receiver operating characteristic curve for binary classification.

Analysis of voice samples can also help in the prediction of depression or anxiety. A study was conducted with 2 sets of participants: one set of participants who had a diagnosis of depression or anxiety and another set of participants who did not have such a diagnosis [39]. Using an app developed for the study called Ellipsis Health, 5-minute voice samples and responses to survey questions were collected from a total of 263 participants (all current patients of a health care clinic) over a period of 6 weeks. Using a model developed with LSTM, the study tested the feasibility of assessing the presence of clinical depression and anxiety by using data from the smartphone app. With a similar approach, another study collected answers to questions in several self-reported psychiatric scales and questionnaires via audio recordings from 124 participants using an Android app developed specifically for the study [40]. Six different algorithms (LR, RF, SVM, XGBoost, KNN, and DNN) were used to study the features generated from audio and to evaluate the results.

In another study, behavioral and physiological data were collected from 212 participants through wearable sensors (including a wristband and a biometric tracking garment) and various surveys to create a dataset of human behaviors. The dataset was then studied to predict the emotional state of the participants [41]. A phone, Unihertz Jelly Pro, was also provided to participants to capture their speech data. An app, TILES, was created to track activities as well as receive responses to surveys. Furthermore, data from other smartphone apps, such as the Fitbit app (to receive updates from the Fitbit wristband), OMsignal app (to record data from the OMsignal smart garment), and RealizD app (to record screen-on time and phone pickups), were also used.

Using only speech data, an automatic depression detection model was developed using deep convolutional neural network (DCNN) [27]. A total of 318 participants (153 diagnosed with major depressive disorder) were asked to record their voices through a smartphone while reading a predefined text. RF, SVM, KNN, and linear discriminate analysis classifiers were used, with RF providing the best accuracy. A similar study was conducted with 163 participants (88 diagnosed with depression), in which speech data were collected using VoiceSense, a voice-collection app installed on each participant’s phone, through vocal responses to 9 general questions [42]. A repeated random subsampling cross-validation method, with random split of the dataset into training and test subsamples and multiple iterative repeats of the process, was used to obtain a predictive equation. Behavioral states of infants can also be predicted by analyzing the audio of their cries. About 1000 cries gathered from 691 infants using the smartphone app ChatterBaby were analyzed and classified into 3 states: fussy, hungry, and pain, using RF [43]. The study also aimed to verify that colic cries may indicate pain and are more similar to pain cries compared with either fussy or hungry cries.

Along with emotional states, it is also possible to create models to predict complex psychiatric conditions, such as schizophrenia, by using data from smartphones. Numerous data were collected from 61 participants, including app usage, reception of calls and SMS text messages, smartphone acceleration data, screen on/off duration, location, speech and conversation, sleep, and ambient environment, and an ecological momentary assessment was performed every 2 to 3 days [44]. Multiple-output support vector regression (m-SVR) and multi-task learning (MTL) with leave-one-out cross-validation were used to train data for each patient and to predict the scores for all possible symptoms.

Audio data can also be used to predict health-related anomalies, such as fatigue level and blood pressure. Using 1772 voice recordings from 296 participants, a model was created to predict fatigue in people affected with COVID-19 [45]. Two types of audio data were collected: recording of participants reading a predefined text and another recording of them pronouncing the vowel “a” for as long as they could. The data were trained and tested using LR, KNN, SVM, and soft voting classifier algorithms. In another study, a stethoscope attached to a smartphone was used to collect heart sound signals from 32 healthy subjects, with the participants laying on a mattress and the stethoscope being placed on their chest [46]. SVM was used for training and testing the estimation model, and 10-fold cross-validation was used to test the accuracy of the model.

Other uses of audio analysis include the inspection of bowel sounds for tracking or predicting digestive diseases [47]. A total of 100 participants were asked to put the smartphone over the lower right and left areas of their abdomen to collect audio via a bowel sound recording app. CNN- and LSTM-based recognition models were developed. For cross-validation, multiple training-test splits were conducted, and 9-fold cross-validation was performed. Furthermore, it is also possible to determine the quality of sleep by analyzing the audio during sleep. Using an app that records audio with the built-in microphones of smartphones and a smart alarm, sleep events, such as snoring and coughing, were identified [48]. SleepDetCNN, a CNN-based model, was created to classify the sleep audio into 3 types: snoring, coughing, and others. Snoring was further studied in 16 patients with habitual snoring tendencies using a smartphone-based gaming app as a treatment for snoring [49]. A section of participants had 15 minutes of daily gameplay (3 voice-controlled games; 5 minutes each), and the majority of participants were provided with microphones to record their sleep for the entire night at least twice per week during the experiment period of 12 weeks. To train the classification models using SVM, 1000 sleep sounds were randomly selected and labeled as “snore” or “not snore” by 2 blinded members of the research team.

In addition to the prediction of diseases, data from smartphone microphones, combined with other data, such as accelerometer, gyroscope, light proximity, and Wi-Fi scan data, have been utilized for emotion prediction [39] as well as the recognition of day-to-day activities [50]. The ADL Recorder app, created for tracking and monitoring the activities of elderly people, recorded both behavioral and contextual data. Various kinds of machine learning classifiers, such as Bayesian network, hidden Markov model, Gaussian mixture model, RF, and KNN, were used throughout for analyzing data from different sensors, and J48 DT was used for the final recognition of activities.

#### Studies Conducted With Both Databases and Experiments

Due to recent global events, numerous experimental studies have been conducted for COVID-19 detection and prediction. Audio data from 497 participants, including those with and without COVID-19, were tested on a model created for analyzing respiratory behavior and compared with a clinical diagnosis [51]. The model, which used LR, was created to train data collected in a study of over 3000 patients diagnosed with asthma and other respiratory diseases. The participants used a smartphone app to send a continuous “aaah” sound spoken for a 6-second duration, along with responses to a questionnaire about any possible symptoms.

In another study, accelerometer and voice recorder data were collected from participants with and without Parkinson disease, and a detection model was created using naive Bayes, KNN, and SVM methods [52]. The same model was used to detect Parkinson disease by using a new dataset obtained from patients newly suspected of having Parkinson disease. They were requested to pronounce the vowel “a” for 10 seconds, and a smartphone was kept at a certain distance (8 cm) from the patients to record the audio.

The dataset from the mPower study [31] was further used to test a voice condition analysis system for Parkinson disease, which was also verified using an experimental dataset (UEX) [53]. Six different machine learning classifiers (LR, RF, GBoost, passive aggressive, perceptron, and SVM) were applied to compare the performance with the 2 different speech databases. For creating the UEX dataset, 60 participants aged between 51 and 87 years were recruited. Of these 60 participants, 30 had Parkinson disease. A smartphone was used to record 3 different samples of the participants pronouncing the “a” vowel continuously without being interrupted.

Voice data from the smartphones of patients with bipolar disorder were studied to determine if it is possible to differentiate people who have bipolar disorder and those who are either unaffected or have any relatives with bipolar disorder [54]. Data from 2 studies, namely the RADMIS trial [55] and the Bipolar Illness Onset (BIO) study [56], were used. The RADMIS trial was conducted with people diagnosed as having bipolar disorder who used a smartphone-based monitoring system installed on their phones, which collected voice data (only of those with Android smartphones) and other smartphone-related data, such as sleep duration and app usage. In the BIO study, participants included those who had bipolar disorder, those who had relatives with bipolar disorder, and those who were not diagnosed with bipolar disorder. The RF model developed from the data was verified using 5-fold participant-based cross-validation.

In some cases, the datasets for training the machine learning models were not obtained from previous studies. Various online sources were used for extracting both the crying and noncrying (eg, talking, breathing, hiccups, and yelling) sounds of infants [57]. For the validation of the algorithm, an independent dataset was created by using real-life recordings of 4 infants at home and 11 infants in a pediatric ward, where the recordings were created using smartphones. RF, LR, and naive Bayes were used for the classification and identification of crying and noncrying sounds.

Similarly, 41 YouTube videos and 5 cough sounds from the SoundSnap website were used to train a cough recognition model [58]. The study also included the development of a smartphone app, HealthMode Cough, that recorded continuous sounds, including sounds from streets, crowded markets, train stations, etc. The recordings were used to test the model, which used DCNN. Another model was created using CNN in a study aimed at analyzing the breathing sounds of participants with the smartphone app Breeze 2 [59]. The dataset for training the model was created using 3 separate datasets: a subset of the dataset from the study by Shih et al [60], which contained breathing sounds; the dataset ESC-50 [61], which contained 50 classes of environmental sounds; and a dataset from 2 participants, which contained a 2-minute breathing training session recorded using a smartphone. For the experiment, 30 participants without any respiratory diseases used the Breeze 2 app to perform 2 breathing sessions for 3 minutes: one with and one without headphones.

A dataset, compiled from multiple sources, was used to train a cough detection model for infants [57]. The cough sounds were obtained from 91 publicly available videos on YouTube consisting of coughing children aged between 0 and 16 years. Noncoughing sounds, such as talking, breathing, cat sounds, sirens, and dog sounds, were obtained via audio clips from YouTube, GitHub, and the British Broadcasting Corporation sound library. Furthermore, the audio data of 21 children, who were admitted with conditions, such as bronchitis, pneumonia, respiratory infection, and viral wheezing, were also collected via an Android smartphone. Using the data of 7 children out of the 21 and adding cough and noncough sounds from different sources, a model was created, and the data from the remaining 14 children were used as a validation dataset. The classification performance of the cough detection algorithm was compared using 2 ensemble DT classifiers: RF and GBoost.

### Research on the Skin

Studies on the use of smartphone features to assess skin-related anomalies have mostly focused on the prediction or identification of skin cancer traits [57,58], and some studies have evaluated the detection of neonatal jaundice [62] and acne [63].

#### Studies Conducted Using Databases

To create a model for predicting skin cancer, 2 sets of databases were used in the study by Dascalu et al [64]: one with dermoscopic images (HAM10000 [65] and Dascalu and David [66]) and another with nondermoscopic images (Pacheco et al [67]). The images were obtained by taking pictures from a digital camera or a smartphone. Comparing the 2 datasets, sensitivity (percentage of correctly diagnosed malignancies) and specificity (percentage of negative diagnoses) were derived. The CNN-based model was found to improve specificity, though it was acknowledged that a significant amount of future work would be needed for improving sensitivity. It was also concluded that the dermoscopic images provided better accuracy compared to those from smartphones.

#### Studies Conducted Through Experiments

Acne is a common skin anomaly, which is experienced by about 10% of the world population. To predict and analyze such skin-related afflictions, many skin image analysis algorithms have been created [63]. To make the analysis and prediction accessible, it would be better to have such a system within a smartphone app. A CNN-based model was developed for acne detection, and an acne severity grading model was developed using the LightGBM algorithm. To test the models, an experiment was conducted, in which 1572 images of the faces of participants were taken from 3 different angles by using iOS or Android smartphones through a smartphone app called Skin Detective, and the dataset was divided in a ratio of 70:30 for training and testing. For ground truth, the images were labeled by 4 dermatologists.

Similarly, for predicting skin cancer, a melanoma detection model was created. A total of 514 patients from dermatology or plastic surgery clinics who had at least one skin lesion were selected, and pictures of their lesions were taken using 3 different cameras: 2 smartphone cameras and 1 digital camera [68]. For the analysis of the experiment dataset, an artificial intelligence algorithm, Deep Ensemble for Recognition of Malignancy [69], developed for determining the probability of skin cancer using dermoscopic images of skin lesions, was used.

Unlike for disease prediction using audio, data for skin-related anomalies can be obtained from other gadgets, such as smart wearables, through which information, such as heart rate, skin temperature, and breathing rate, can be obtained. A combination of data from smartphones (smartphone-based social interactions, activity patterns, and number of apps used) and smartwatches (E4 Empatica; skin temperature) obtained via the in-house smartphone app MovisensXS was used to predict emotional changes and the severity of depression in people [70]. The study was conducted over a period of 8 weeks and included 41 people with depressive disorder. The participants had to complete daily smartphone-delivered surveys, a clinician-rated symptom assessment test, and a blood test to screen for potential medical contributors of depressed mood.

#### Studies Conducted With Both Databases and Experiments

Neonatal jaundice is a frequently occurring condition, which can also be diagnosed using smartphone images [62]. A study was conducted with 100 children, aged between 0 and 5 days, in which a picture or video was taken of their full face, with a calibration card, to capture their skin and eye sclera. Ground truth was established by noting their transcutaneous bilirubin (TCB) level, and the pictures were labeled “jaundiced” or “healthy” by a pediatrician. A CNN-based model was trained using the ImageNet dataset [71] and was used to test neonatal jaundice tendencies using the experiment dataset and transfer learning. Multilayer perceptron (MLP), SVM, DT, and RF were also used for diagnosis, where it was determined that transfer learning methods performed better for skin features, while machine learning models performed better for eye features.

### Research on the Eye

Diabetic retinopathy was the most commonly studied disease [66,67,69] among the collected literature for eye-related predictions, along with other varying topics, such as eye tracking, vision monitoring, jaundice, and autism.

#### Studies Conducted Using Databases

An optimized hybrid machine learning classifier with the combination of neural network (NN) and DCNN with a single-stage object detection (SSD) algorithm was proposed to be used with the retinal images taken from a smartphone-enabled DIY camera [25] to predict diabetic retinopathy. Since there was a scarcity of image data captured using DIY smartphone-enabled devices, the model was validated with analysis of 2 other databases that contained fundus images: APTOS (2019 blindness dataset) and EyePACS, and the model performed better in comparison to the individual results of the NN, DCNN, and NN-DCNN methods.

CNN-based models usually tend to provide the best performance in image recognition tasks. With that in mind, the APTOS (2019 blindness dataset) and EyePACS datasets were used to build a CNN-based model for predicting diabetic retinopathy [72]. The algorithm was then externally validated using the Messidor-2 dataset [73], which contained about 1058 images from 4 French eye institutions. The algorithm was further tested on the EyeGo dataset, which contained 103 fundus images from 2 previously published studies obtained by using an EyeGo lens attachment and an iPhone.

#### Studies Conducted Through Experiments

Almost 51% of eye diseases in the United States are related to cataract [74]. It will be convenient to use images from smartphones for the early detection of cataract, and the results will be provided instantly. By taking pictures with a smartphone camera, 100 samples were collected from participants (50% of the participants had cataract) [74]. SVM was applied on the dataset, and the accuracy was 96.6% for cataract detection.

In addition to images of the eye, videos of eye movement can be used for different kinds of diagnoses, such as for autism, since atypical eye gaze can be considered as an early symptom for autism spectrum disorder (ASD) [75]. The behaviors of 1564 toddlers were recorded using the front camera of an iPhone or an iPad when the toddlers, accompanied by their caregivers, viewed engaging movies for less than 60 seconds on the device. Using computer vision analysis on the data, it was found that children with ASD have less coordinated gaze patterns while viewing movement in movies or following conversation between 2 moving people.

In addition to the in-situ collection of data, smartphones can be used for remote collection of data. To determine the attention span of infants by tracking their gaze, an online webcam-linked eye tracker called OWLET was developed, and experiments were conducted with 127 infants remotely [76]. The infants were in the presence of their caregivers, who used either a smartphone or a computer to access the tracking task and provided their responses of the infant behavior using a questionnaire. For the experiment, a video (an 80-second Sesame Street video) was played, and the eye movements of the infants were recorded, tracked, and analyzed. No difference was found in the image data between the smartphone and computer, which was verified by a 2-sided independent samples *t* test and chi-square test. LR was used to examine the efficiency of the OWLET system.

Similarly, 417 adults with active or passive vision-related problems took part in an experiment using a smartphone app named Home Vision Monitor (HVM) to self-test their vision [77]. The app required them to submit an eye vision test twice per week, and their smartphone usage and app usage history were recorded by the researchers. RF and LR were used for statistical analysis.

The app EyeScreen was developed to support retinoblastoma diagnosis for the presence of leukocoria [78]. About 4000 eye images were obtained from about 1460 participants via the app, and an ImageNet model, ResNet, was used for image processing by dividing the dataset in an 80:20 ratio for training and testing. The app had the feature to process the image within it and provide the result.

#### Studies Conducted With Both Databases and Experiments

A common anomaly, neonatal jaundice, was investigated [62], for which a dataset of healthy and jaundiced individuals was created in an experiment conducted over 35 to 42 weeks. In the experiment, a full-face photo was clicked to capture the eye sclera. To obtain ground truth data, the TCB level was measured using a jaundice meter device, and the pictures were labeled “jaundiced” or “healthy” by a pediatrician.

Tracking eye movements has been a topic of interest for a wide variety of research ranging from autism [75] and tourism [28] to driving and gaming [79]. A multi-layer feed-forward convolutional neural network (ConvNet) model was created and trained on the GazeCapture dataset [80], which was created from the data of 1474 participants using an iPhone or iPad. To verify the model, an experiment was conducted using a custom-made Android app, in which eye gaze videos were captured using the front facing camera of the phone. The participants were asked to follow a stimulus on the mobile screen, which could be a dot or movement of a circular, rectangular, or zig-zag pattern.

Two sets of experiments were carried out, with one using a smartphone (iPhone 6) and another using smartphone-based retinal imaging systems, such as iExaminer, D-Eye, Peek Retina, and iNview [81], to create a model for the diagnosis of diabetic retinopathy. The CNN-based AlexNet architecture was used for transfer learning, which was first trained using 1234 images from the EyePACS dataset. Then, the architecture was tested with 138 retinal images from datasets, including those of the EyePACS, iExaminer, D-Eye, Peek Retina, and iNview systems.

### Exclusion of Papers

A total of 11 papers were excluded from the final selection after reviewing the full text of all the papers using the selection criteria. Among them, 5 were excluded because the studies did not involve the use of smartphones for data collection. Similarly, 3 of the papers passed the initial screening test because of the presence of words, such as smartphone, eye, and audio, in their abstract. However, the studies were not relevant to the topic of our review. Furthermore, 2 of the studies were excluded because they only included the proposal of the method of disease prediction using machine learning and smartphones. Finally, a paper was excluded as it included a discussion about the topic but did not contain any database analysis or experiment. Among the papers that provided a proposal of a disease prediction system, it is worth mentioning that the paper by Bilal et al [82] was very detailed and well explained.

**Figure 2:**
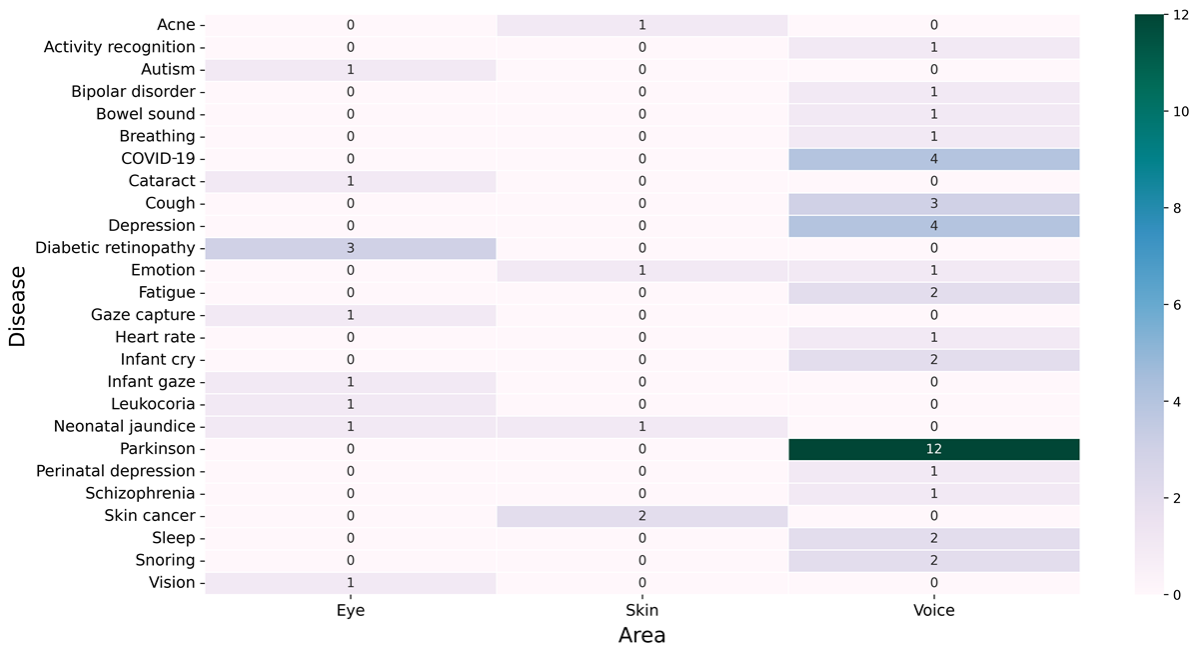
Diseases studied in the collected papers.

After the full-text screening of papers, there were 34, 5, and 10 relevant papers in the categories of voice, skin, and eye, respectively. These studies were further analyzed by focusing on the diseases dealt with in each study and the different health topics. The results can be seen in Figure 2. Parkinson disease was the most studied (n=12) disease among the collected studies, followed by COVID-19 (n=4), depression (n=4), cough (n=3), and diabetic retinopathy (n=3). It can be argued that cough and COVID-19 could be included under the same category and depression and emotion could be included under the same category. However, based on the terminologies and methods used in the papers, we have treated them separately.

## Discussion

### Overview

The use of technology in the medical field has seen massive growth in recent years. A lot of improvements have been made in different areas, such as handling complex electronic medical records [83], and in identifying and predicting various diseases, such as lung anomaly detection using computed tomography scan images [84], emotion detection using different data from smartphones [35], and identification of the burden faced by people who travel to separate locations for receiving health care services [85]. Smartphones provide a low-power, small-sized, and easy method for data collection and analysis, which differs from the usual bulky, expensive, and complex systems used for biomedical data collection and analysis.

Smartphones are equipped with numerous sensors and high-quality cameras, making it easy to collect different types of data. Moreover, due to the COVID-19 outbreak and the changes in the overall working environment that followed, there has been a strong focus on delivering health services remotely [86]. The disease identification process can be made efficient by using smartphones to collect data and provide a diagnosis, as well as deliver results to patients.

With these factors in mind, we focused on research carried out using machine learning and data from smartphones to identify or predict diseases. We selected 3 areas to focus on and formulated the research questions. We conducted a review of the available papers collected using the screening method explained in the section Study Selection. Here onwards, we will discuss the results for our research questions.

### Research Question 1: What Are the Databases Available for Eye, Skin, and Voice Analysis?

We found a total of 31 databases in the collected studies, including an unclear source, vaguely referred to as “online sources” [57]. In most of the cases, the databases were used to create a model for disease prediction. However, there were also instances where the databases were used to validate a model developed using experimental data [81] or using other databases [72]. Since the number of collected voice-related studies was higher than that of skin- or eye-related studies, a similar difference in number can be observed for the list of databases, as shown in Tables 5-7. The numbers of databases for voice, skin, and eye were 22, 4, and 5, respectively. The voice-related databases were used to predict a variety of diseases or health statuses, such as Parkinson disease [26,29], emotion [35], bipolar disorder [55], and infant cry [57]. Owing to the COVID-19 pandemic, many databases were used for the detection of COVID-19 or cough-related anomalies [20,24,46,52]. Of 4 skin-related databases, 3 were aimed at the prediction of skin cancer [64] and the remaining database was related to neonatal jaundice [62]. The same database by Althnian et al [62] was also used for jaundice detection using retinal images. Diabetic retinopathy was the most common disease among eye databases [66,67,69]. Eye databases also consisted of data related to capturing eye movement [80] or gaze/concentration [74].

**Table 5:** Databases with voice data.

Database	Frequency
CoughVid	1
TASK	1
Brooklyn	1
Wallacedene	1
GoogleAudio dataset	1
Freesound	1
Librispeech	1
Coswara	2
ComparE	1
Sarcos	1
mPower	4
PC-GITA	1
Vishwanathan	1
ExtraSensory	1
Online sources	3
Shih et al [60]	1
UEX	1
RADMIS	1
Bipolar Illness Onset	1
YouTube	3
SoundSnap	1
BBC sound library	1

**Table 6:** Databases with skin data.

Database	Frequency
Ham10000	2
ImageNet	1
Dascalu and David [66]	1
Pacheco et al [67]	1

**Table 7:** Databases with eye data.

Database	Frequency
Messidor-2	1
GazeCapture	1
EyePacs	1
APTOS	1
EyeGO	1

### Research Question 2: What Are the Machine Learning Models Used for Eye, Skin, and Voice Analysis?

Similar to databases, machine learning models were also used either in separation [67,87] or as a comparison along with multiple other models [6,24,31], and sometimes as ensemble classifiers [20,62]. As the same study usually consisted of multiple machine learning methods, the frequency of use of certain machine learning methods was considerably high. To investigate the best machine learning method for each kind of data, instead of using numbers, we calculated the frequency of use of a particular machine learning method for each of the 3 areas. We were then able to determine the rate of machine learning methods for each area, as shown in Tables 8-10. The most common machine learning method used for voice-related data was RF, while CNN was the most used for both eye- and skin-related data.

**Figure 3:**
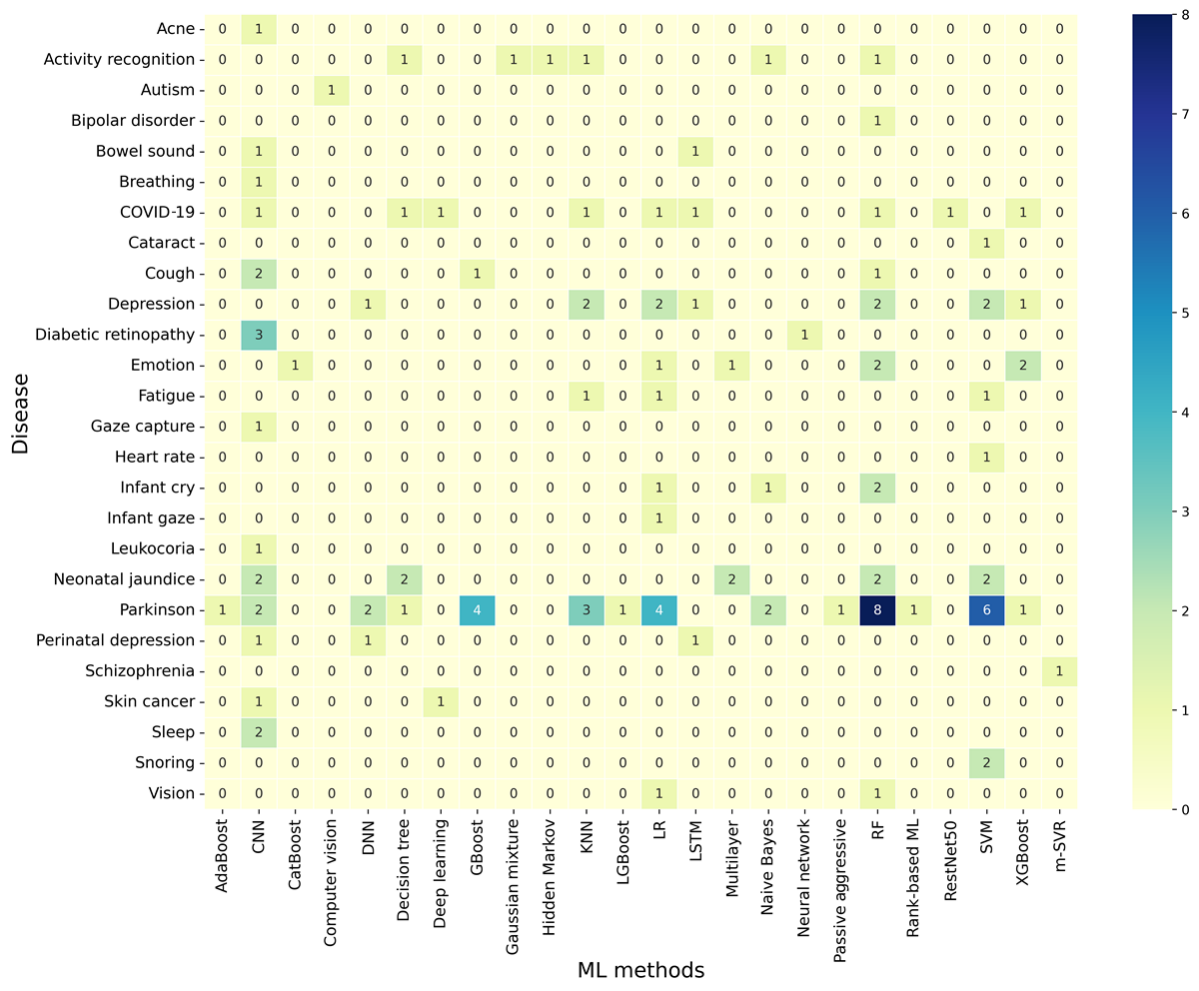
Use of machine learning (ML) methods based on the type of disease. AdaBoost: adaptive boosting; CNN: convolutional neural network; DNN: deep neural network; GBoost: gradient boosting; KNN: k-nearest neighbor; LGBoost: light gradient boosting; LR: logistic regression; LSTM: long short-term memory; m-SVR: multiple-output support vector regression; RF: random forest; SVM: support vector machine; XGBoost: extreme gradient boosting.

For further analysis, we expanded on the diseases and determined the frequency of the use of each machine learning method for each of the diseases or anomalies found in the collected papers. The results are shown in Figure 3. The figure shows all machine learning methods used across various studies for each of the diseases. Since many studies used multiple machine learning methods (especially for Parkinson disease), the frequency of use of some methods, such as RF, SVM, CNN, and LR, was high.

**Table 8:** Machine learning methods used with voice data.

Machine learning method	Rate of use, %
AdaBoost^a^	1.1
CNN^b^	10.9
CatBoost	1.1
DNN^c^	4.3
Decision tree	3.3
Deep learning	1.1
GBoost^d^	5.4
Gaussian mixture	1.1
Hidden Markov	1.1
KNN^e^	8.7
LGBoost^f^	1.1
LR^g^	10.9
LSTM^h^	4.3
Multilayer	1.1
Naive Bayes	4.3
Passive aggressive	1.1
RF^i^	18.5
Rank-based machine learning	1.1
RestNet50	1.1
SVM^j^	13.0
XGBoost^k^	4.3
m-SVR^l^	1.1

^a^AdaBoost: adaptive boosting.

^b^CNN: convolutional neural network.

^c^DNN: deep neural network.

^d^GBoost: gradient boosting.

^e^KNN: k-nearest neighbor.

^f^LGBoost: light gradient boosting.

^g^LR: logistic regression.

^h^LSTM: long short-term memory.

^i^RF: random forest.

^j^SVM: support vector machine.

^k^XGBoost: extreme gradient boosting.

^l^m-SVR: multiple-output support vector regression.

**Table 9:** Machine learning methods used with skin data.

Machine learning method	Rate of use, %
CNN^a^	30.0
Decision tree	10.0
Deep learning	10.0
Multilayer	10.0
RF^b^	20.0
SVM^c^	10.0
XGBoost^d^	10.0

^a^CNN: convolutional neural network.

^b^RF: random forest.

^c^SVM: support vector machine.

^d^XGBoost: extreme gradient boosting.

**Table 10:** Machine learning methods used with eye data.

Machine learning method	Rate of use, %
CNN^a^	41.20
Computer vision	5.88
Decision tree	5.88
LR^b^	11.76
Multilayer	5.88
Neural network	5.88
RF^c^	11.80
SVM^d^	11.80

^a^CNN: convolutional neural network.

^b^LR: logistic regression.

^c^RF: random forest.

^d^SVM: support vector machine.

### Research Question 3: How Are the Data Collected From Smartphones?

To collect audio-related data, the built-in smartphone microphone was used most of the time, both at home [26,37] and in the experimental set up [52]. In some cases, external microphones were also used [36]. Similarly, in many cases, audio was also collected via custom-made smartphone apps [21,34,38], and in some cases, it was collected via a web interface that could be accessed using smartphones [20,25].

For the collection of skin data, pictures and videos were mainly taken with a smartphone [58,59]. In some cases, smartphone apps were also created for data collection [60,63]. Frequently, pictures from smartphones were not considered adequate for taking retinal images, and an external lens or retinal imaging system was used alongside the smartphone to collect eye data [20,66,67]. However, experiments have also shown that smartphone images are equally effective to analyze eye-related anomalies [25,71]. For collecting gaze-related data, videos taken from the front camera of smartphones have been used effectively [75,81].

Data of both the skin and voice have been used for the detection of emotion and depression. However, in such studies, data apart from voice and skin data were also collected. Combinations of data from smartwatches, such as heart rate, skin temperature, and breathing rate, and data from smartphones, such as smartphone-based social interactions, activity patterns, and the usage of apps, were used for detecting emotional changes and the severity of depression [70]. Similarly, for detecting emotional status, voice and other data, such as location, accelerometer data, gyroscope data, and phone usage, were used [31,36]. For the detection of Parkinson disease, data apart from voice data, such as gait and balance [26], tapping of buttons on a smartphone screen [26,33], and accelerometer data [52], were collected. Similarly, in a study for vision monitoring, data apart from images of the eyes, such as phone and app usage, were collected using a smartphone app [77]. Furthermore, in many studies, surveys and questionnaires were also regularly received from participants during data collection, especially via smartphone apps [26,34,36,63].

It is worth noting that there are many sensors available in smartphones, such as accelerometers and gyroscopes, which can be helpful in determining the speed of touch, posture, walking speed, location, etc. Therefore, even if the aim is to analyze a particular health area, the same combination of data, collected from multiple data sources, can be used to identify different diseases. For example, in the study of Parkinson disease, data, such as voice, accelerometer data, location, application usage, and other phone data, were usually collected. The same data can also be analyzed to detect emotional changes or depression. Similarly, when collecting data on skin abnormalities, it is possible to obtain facial data that can also be used for eye-related analysis.

Moreover, it has been observed that with remote health monitoring systems, especially with the use of smartphones, people often have concerns over the access and use of their data, such as location, application usage, screen time, and browsing history [78,79]. These concerns of smartphone apps are higher as they contain sensitive behavioral data. To tackle these issues, it is necessary to build trust with the users regarding the app and its data collection methods. It was found that state-funded research institutes had higher levels of trust with people compared to private institutions [88]. This shows that to conduct research using smartphones and gather user data, it is necessary to involve trusted institutions for governing the study, as well as have transparency over data collection, distribution, and use of the results.

### Limitations

There are some limitations. First, for screening the collected papers based on their titles and abstracts, we used a keyword screening method [23]. Although great care was taken in the selection of keywords for this screening, it must be acknowledged that some papers may have been overlooked if they did not contain the specified keywords. We firmly believe that such a limitation can occur, but the number of studies will be very few. Second, we focused only on studies that used smartphones. This could lead to the exclusion of recent studies that did not consider the use of smartphones to collect health-related data.

Moreover, we only selected studies that analyzed eye-, skin-, and voice-related diseases. Because of the niche approach of this scoping review, we did not consider a lot of other health areas where smartphones might have been used to gather data for machine learning analysis. Furthermore, many new machine learning models and other algorithms are being developed, and existing algorithms are being improved [89]. These methods have not been used but could potentially be used for health diagnosis, and thus, they have been overlooked in this review.

### Overall Summary

The field of the use of machine learning on smartphone-obtained data for health care purposes is ever evolving. Through this study, we aimed to provide information about studies that have conducted experiments related to eye-, skin-, or voice-related diseases, where data were obtained strictly via smartphones. Similarly, we have provided details of publicly available databases that have been used in studies to apply machine learning methods for developing models to predict eye-, skin-, or voice-related diseases. Researchers working in similar fields can use the experiment details or the databases presented in this study to design their research. Furthermore, the machine learning model to use for a study needs to be determined with much consideration. We have presented machine learning models applied based on the study area as well as the types of diseases. Therefore, the information provided in the paper can help reduce the time and effort for researchers in designing experiments and selecting the databases or machine learning models to use in their studies. Our title and abstract screening method is also easy to understand and replicate, and could be used by researchers aiming to perform scoping reviews or systematic literature reviews.

### Conclusion

There has been a growth in the number of studies based on the application of machine learning methods to data obtained from smartphones for the prediction of diseases. However, there are few literature reviews that provide information about the databases used, experiments carried out, and machine learning methods applied. We formulated a scoping review to identify the studies that have been conducted, specifically related to the 3 areas of skin, eye, and voice, and determined the studies that conducted experiments using smartphones to gather skin-, eye-, and voice-related data; the publicly available databases that include skin, eye, or voice data; and the machine learning methods that are commonly implemented in such studies. Furthermore, with this research, we intended to test the effectiveness of the keyword screening method that we developed. We first searched for relevant studies and screened them by applying our keyword screening method to their titles and abstracts. We analyzed the full text according to the inclusion and exclusion criteria and collected a total of 60 studies.

After assessing the full text of all identified studies, we discarded 11 studies, and among the remaining 49 studies, we found 24 different machine learning methods and 31 different databases used. The details from these collected studies provide insights into how the experimental studies were conducted, which databases were used, and which machine learning methods provided better results. The relevance and quality of the information acquired proved that our keyword screening method was effective in screening papers relevant to the topic and thus could be adopted by researchers for conducting scoping reviews. The use of our results can help reduce the time and effort required by researchers working in the field of artificial intelligence for health care to gather such information in detail. Moreover, the results presented can be used to select databases for future studies, replicate the experimental design, or select machine learning models suitable for the topic of interest.

## Appendix

Multimedia Appendix 1 PRISMA (Preferred Reporting Items for Systematic Reviews and Meta-Analyses) checklist for scoping reviews.

## Abbreviations

ASD: autism spectrum disorder
BIO: Bipolar Illness Onset
CNN: convolutional neural network
DCNN: deep convolutional neural network
DNN: deep neural network
DT: decision tree
GBC: gradient boosting classifier
GBoost: gradient boosting
KNN: k-nearest neighbor
LR: logistic regression
LSTM: long short-term memory
NN: neural network
RF: random forest
SVM: support vector machine
TCB: transcutaneous bilirubin
XGBoost: extreme gradient boosting
